# Tolerance of fungal infection in European water frogs exposed to *Batrachochytrium dendrobatidis* after experimental reduction of innate immune defenses

**DOI:** 10.1186/1746-6148-8-197

**Published:** 2012-10-23

**Authors:** Douglas C Woodhams, Laurent Bigler, Rachel Marschang

**Affiliations:** 1 Institute of Evolutionary Biology and Environmental Studies, University of Zurich, Winterthurerstrasse 190, Zurich, CH-8057, Switzerland; 2 Institute of Organic Chemistry, University of Zurich, Winterthurerstrasse 190, Zurich, CH-8057, Switzerland; 3 Institute for Environmental and Animal Hygiene, University of Hohenheim, Garbenstrasse 30, Stuttgart, 70599, Germany; 4 Department of Ecology and Evolutionary Biology, University of Colorado, N122 Ramaley, 334 UCB, Boulder, CO, 80309-0334, USA

**Keywords:** Amphibian, Antimicrobial peptide, Chytridiomycosis, MALDI-MS, Microbiota, *Pelophylax*, Ranavirus

## Abstract

**Background:**

While emerging diseases are affecting many populations of amphibians, some populations are resistant. Determining the relative contributions of factors influencing disease resistance is critical for effective conservation management. Innate immune defenses in amphibian skin are vital host factors against a number of emerging pathogens such as ranaviruses and the amphibian chytrid fungus *Batrachochytrium dendrobatidis* (*Bd*). Adult water frogs from Switzerland (*Pelophylax esculentus* and *P. lessonae*) collected in the field with their natural microbiota intact were exposed to *Bd* after experimental reduction of microbiota, skin peptides, both, or neither to determine the relative contributions of these defenses.

**Results:**

Naturally-acquired *Bd* infections were detected in 10/51 *P. lessonae* and 4/19 *P. esculentus*, but no disease outbreaks or population declines have been detected at this site. Thus, this population was immunologically primed, and disease resistant. No mortality occurred during the 64 day experiment. Forty percent of initially uninfected frogs became sub-clinically infected upon experimental exposure to *Bd.* Reduction of both skin peptide and microbiota immune defenses caused frogs to gain less mass when exposed to *Bd* than frogs in other treatments. Microbiota-reduced frogs increased peptide production upon *Bd* infection. Ranavirus was undetectable in all but two frogs that appeared healthy in the field, but died within a week under laboratory conditions. Virus was detectable in both toe-clips and internal organs.

**Conclusion:**

Intact skin microbiota reduced immune activation and can minimize subclinical costs of infection. Tolerance of *Bd* or ranavirus infection may differ with ecological conditions.

## Background

Experimental studies are needed in disease ecology to determine the relative importance of factors influencing disease outcome. Results can be applied to conservation management of amphibians encountering a variety of infectious diseases emerging with global change [1-5]. Two predominant amphibian pathogens associated with global population declines are ranaviruses and the amphibian chytrid fungus *Batrachochytrium dendrobatidis* (*Bd*).

Ranaviruses are large icosahedral DNA viruses belonging to the family Iridoviridae. They have been detected in fish, amphibians and reptiles. Ranavirus associated disease in amphibians has been reported in the Americas, Europe, and Asia, and ranaviruses have also been detected in amphibians in Australia [4,6]. To our knowledge, there are no previous reports of ranavirus occurring in wild-caught amphibians in Switzerland.

*Bd* is the causal agent of chytridiomycosis, an amphibian disease capable of producing epizootics and perhaps species extinctions [3,7]. The fungus is top-ranking in the most recent Amphibian Conservation Action Plan [2]. In Europe, the earliest archived specimens with *Bd* infections were found in 1998 [8], however, recent studies have detected the fungus in Switzerland from much older samples (N. Peyer and B. Schmidt, unpublished data), and both a Swiss strain and a “global panzootic lineage” have been identified in Switzerland ( [9]; M.C. Fisher, pers. comm.). Infection of wild amphibians has been documented in ten European countries and appears to be widespread ( http://www.Bd-maps.net/). As in other regions, the disease affects some host species more than others [8,10]. Therefore, attention to disease resistance mechanisms may lead to protective interventions for threatened amphibian species.

In Switzerland, many amphibian populations have declined in recent years, and 70% of the 20 species are on the Swiss Red List of threatened species [11]. In contrast, few declines have been reported for populations of water frogs, *Pelophylax lessonae* and *P. esculentus*. These species also appear to be relatively tolerant of *Bd* in the wild [8]. Amphibian host mechanisms of disease resistance include a variety of behavioral, innate, and adaptive immune responses [12]. Symbiotic bacteria associated with amphibian skin can inhibit *Bd* and disease development in some species [13-16]. Antimicrobial peptides (AMPs) may also be important against chytridiomycosis [10,17]. Nearly 50 skin AMPs were previously described from the water frog complex [18-23], and given the similarity of peptide families, some are probably effective against *Bd*[24]. A unique aspect of this study compared to most previous *Bd*-exposure experiments [25], is that adult frogs are examined upon capture from an abundant wild population with their immunity primed, as opposed to examining pathogen-naïve metamorphs. Therefore, adult water frogs are ideal for experiments to test the mechanisms of disease resistance by reducing components of the innate immune system that may confer protection in the wild.

## Results

### Ranavirus infection

Two frogs died within a week of collection. Ranaviruses were deteced by PCR in the liver, kidney, and toe clip of one *P. esculentus* and in the liver of one *P. lessonae*. Virus was isolated on IgH2 from all of the organs tested from both frogs (liver, kidney, heart, lung, and intestine of the first and liver, kidney, heart, and intestine of the second frog). Cell culture supernatant from all isolates was positive for ranavirus by PCR. Sequencing of the PCR products from the livers of each of the frogs showed that a 488 bp portion of the MCP gene was 100% identical between the two viruses and 97.57% identical to the corresponding portion of the genome of FV3, the type species of the genus *Ranavirus*.

### Bd infection

**Figure 1:**
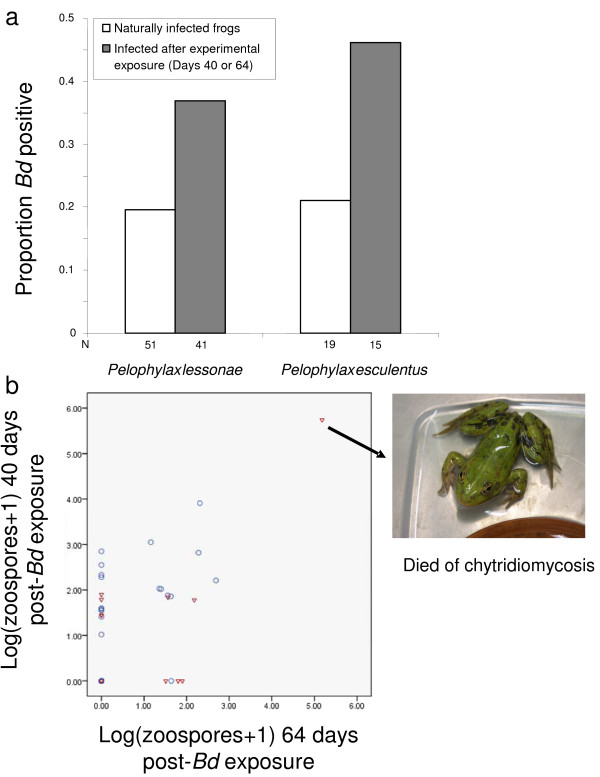
**Infection with *B. dendrobatidis *(*Bd*).** (**a**) Proportion of water frogs infected with *Bd* determined by quantitative rtPCR. (**b**) Infection intensities indicating *Bd* zoospore equivalents measured from swabs of *Pelophylax esculentus* and *P. lessonae* on day 40 post-exposure (feet swabbed) or on day 64 post-exposure (body swabbed after rinse). Frogs that were naturally infected with *Bd* (triangles) or uninfected before beginning the experiment (circles) are indicated. One *P. esculentus* died on day 80. This frog was naturally infected, and in addition to the highest *Bd* infection intensity, clinical signs indicative of chytridiomycosis included progressive shedding of skin in water, splayed limbs, inappetence, and lethargy.

**Figure 2:**
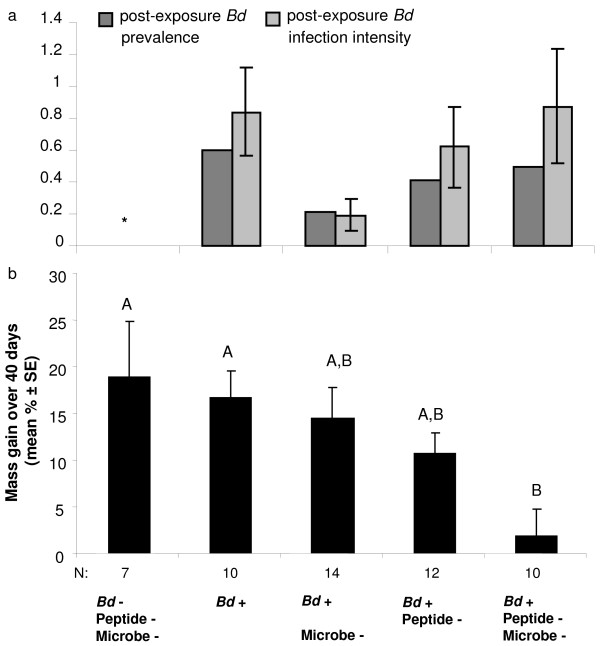
**Change in *B. dendrobatidis *(*Bd*) infection status and mass of initially uninfected frogs (*Pelophylax esculentus *and *P. lessonae*, N = 53) in each experimental treatment.** (**a**) Proportion of frogs that became infected during the experiment and mean (± SE) infection load expressed as log(zoospore equivalents + 1). Asterisk indicates infection was detected in one control frog that may have had an undetectable infection before the experiment. (**b**) Mean percent (± SE) mass gain of frogs in each treatment. Letters above bars indicate homogeneous subsets based on a Tukey post-hoc test.

*Bd* was prevalent in field collected water frogs. Swabs were *Bd* positive for 10/51 *P. lessonae* and 4/19 *P. esculentus* before experimental treatments (Figure 1a). About 40% of initially uninfected frogs of both species developed skin infections upon experimental exposure with *Bd* (Figure 1a). Prevalence before or after treatments was not significantly different between species (Fisher’s exact tests, *P*’s >0.05). These infections were sometimes not detected on feet swabs but were detected on body swabs and vice versa (Figure 1b). The proportion of frogs that became infected in each treatment was not significantly different for *P. lessonae* or both species combined (Pearson Χ^2^, *P*’s > 0.05; Figure 2a). The zoospore infection load did not differ significantly among treatments for *P. lessonae* or both species combined (Kruskal-Wallis tests, *P*’s > 0.05). No mortality occurred during the 64 d experiment. One naturally infected *P. esculentus* died shortly after the experiment (d 80) showing clinical signs of chytridiomycosis and high *Bd* infection loads (Figure 1b).

### Mass change

Nearly all frogs gained mass during the experiment (Figure 2b). Results were similar for both species, thus, the combined results are reported here. Frogs that became infected during the experiment gained slightly, but not significantly, less mass than frogs that remained uninfected (Independent t-test, t_51_ = −0.401, *P* = 0.690). However, frogs that were initially infected in the field (N=14) gained a mean of 1.2% body mass in comparison with initially uninfected frogs (N=53) that gained 12.1% body mass (Independent t-test, t_65_ = −2.992 *P* = 0.004). Initially uninfected frogs that were exposed to *Bd* during the experiment (N=46) gained 11.1% body mass, and initially uninfected frogs that became infected during the experiment (N=20) gained 11.3% body mass. Frogs that were exposed to *Bd* and had both skin peptides and microbiota reduced, gained significantly less weight than frogs in some other treatments, including immune reduced frogs that were not experimentally exposed to *Bd* (Figure 2b; ANOVA, F_4,52_ = 3.612, *P* = 0.012). Thus, *Bd* infection and immune reduction interacted to produce a growth reduction effect.

### Skin peptide recovery

**Figure 3:**
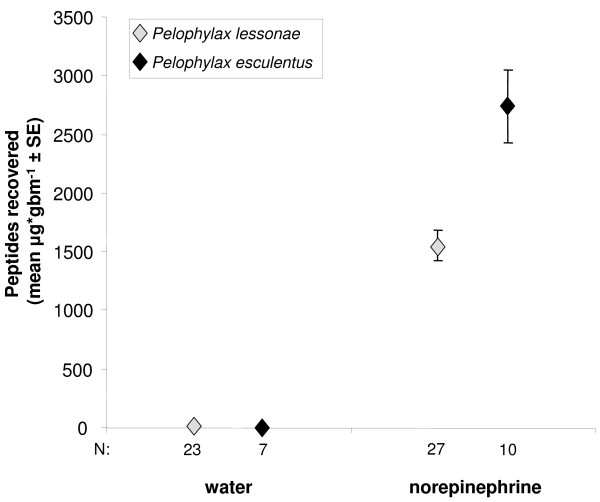
**Skin peptide recovery from *Pelophylax esculentus *and *P. lessonae *upon administration of norepinephrine or water control at the initiation of the experiment.**

**Figure 4:**
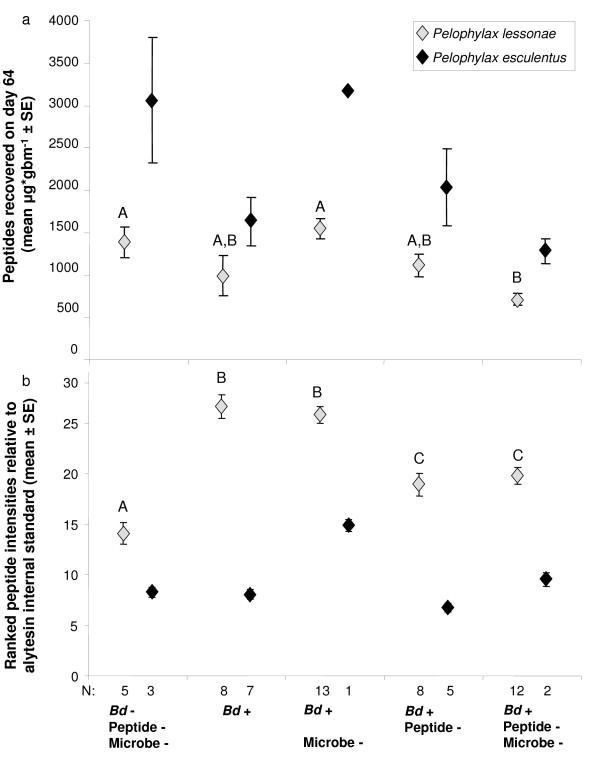
**Quantity and relative intensity of skin peptides recovered from *Pelophylax esculentus *and *P. lessonae *in experimental treatments.** (**a**) Peptide quantities recovered from frogs in each treatment at the end of the experiment indicating that frogs in peptide reduced treatments had recovered peptide stores by 64 days (compare to Figure 3). (**b**) Comparison of mean skin peptide intensities of samples collected from frogs at the end of each experimental treatment. Letters above bars indicate homogeneous subsets based on a Tukey post-hoc test within *P. lessonae*. Small sample size for *P. esculentus* precluded statistical analysis.

The dry weight quantity of peptides recovered after norepinephrine administration was significantly correlated to body mass (gbm) for both species (Pearson correlations, *r* = 0.489, *n* = 27, *P* = 0.0048, *P. lessonae*; *r* = 0.753, n = 11, *P* = 0.0038 *P. esculentus*). Correcting for surface area did not significantly improve this correlation; therefore, we used μg gbm^-1^ as the unit of peptide quantity recovered. Norepinephrine administration induced significant quantities of skin peptides from both species in comparison with water-injected controls (Mann–Whitney U-tests, *P*’s < 0.001; Figure 3). The quantity recovered from controls was (mean ± SD) 5.0 ± 11.7 μg gbm^-1^ from *P. lessonae* and 14.7 ± 13.3 μg gbm^-1^ for *P. esculentus*. *P. esculentus* produced more peptides on average (2741 μg gbm^-1^) than *P. lessonae* (1549 μg gbm^-1^) when stimulated with norepinephrine (Independent t-test, *t*_35_ = −4.030, *P* = 0.0003; Figure 3). Peptide-reduced frogs had recovered peptide quantities similar to non-reduced frogs by 64 days after treatment (Figure 4a). The quantities of peptides recovered on day 64 differed among treatments (Figure 4a). Small sample sizes prohibited testing the treatment effect on *P. esculentus* peptides, but *P. lessonae* with both peptide and microbiota immune defenses reduced and exposed to *Bd* had significantly less peptides than frogs in some other treatments (ANOVA, *F*_4,47_ = 6.043, *P* = 0.0006, Figures 3, 4a).

### Relative intensities of peptides

In addition to the overall quantity of peptides recovered, we measured the relative intensity of peptides within the mass spectra. Mean peptide intensities varied among treatments. In *P. lessonae*, *Bd* exposure and *Bd* exposure with peptide reduction showed the highest intensities, and the control immune-reduced treatment the lowest (ANOVA, *P* < 0.0001; Figure 4b). For *P. esculentus*, small sample sizes precluded statistical testing. Dry weight of partially-purified peptides was significantly correlated to the sum relative intensity of peptide peaks identified by MALDI-TOF MS (Pearson correlation, *r* = 0.376, *n* = 62, *P* = 0.003).

### Peptide production stimulated by infection with Bd

**Figure 5:**
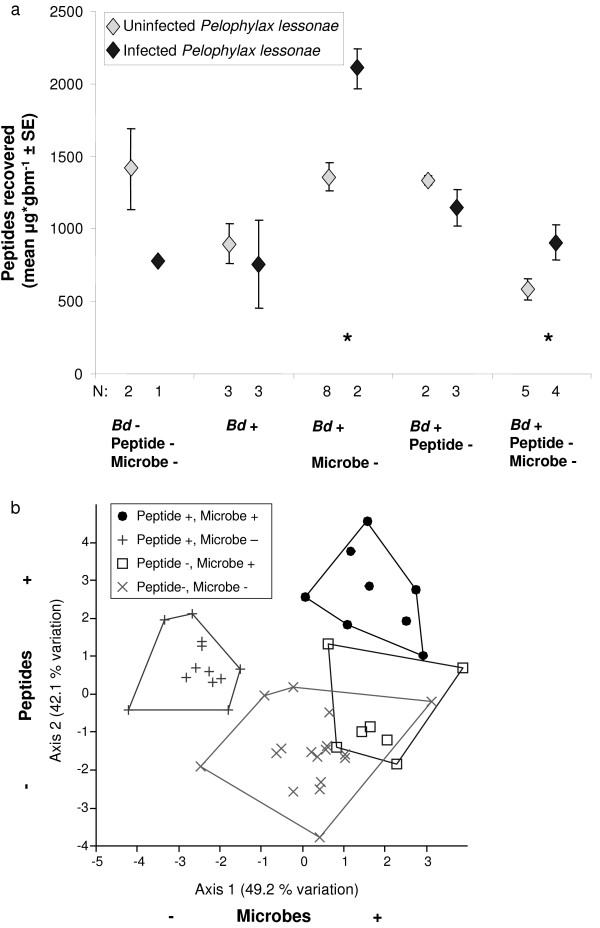
**The effect of infection with *B. dendrobatidis *(*Bd*) on the quantity of skin peptides recovered and the effect of treatment on skin peptide profiles from *Pelophylax lessonae*.** (**a**) Asterisks indicate treatments with statistically significant differences between infected and uninfected frogs. (**b**) A canonical variates analysis scatter plot showing treatment effects on skin peptide profiles. Axes correspond to frog treatment: Peptides reduced (−) by norepinephrine to intact (+), and microbes reduced (−) by antibiotics to intact (+).

In *P. lessonae*, *Bd* infection significantly increased the quantity of peptides recovered from two treatments: *Bd* exposed with microbes reduced, and *Bd* exposed with both microbes and peptides reduced (Independent t-tests, *P*’s < 0.05). Thus, an interaction of reduced microbes and *Bd* exposure increased skin peptide production (Figure 5a).

### Peptide profiles differed among treatment groups

The profiles of *P. lessonae* skin peptide ranked intensities were independently analyzed for the effects of *Bd* exposure, *Bd* infection, norepinephrine treatment, and antibiotic treatment with multivariate analyses of variance. Neither *Bd* exposure, *Bd* infection pre-treatment, nor *Bd* infection post-treatment significantly affected peptide profiles (MANOVA, *P*’s > 0.05). Reducing peptides with norepinephrine significantly affected the profiles of recovered peptides (Wilks’ Lambda, *F*_22,21_ = 2.595, *P* = 0.016). Similarly, reducing microbiota with antibiotics significantly affected peptide profiles (Wilks’ Lambda, *F*_22,21_ = 2.083, *P* = 0.049). When allocated to one of these four treatment combinations (peptides and/or microbiota reduced or intact) differences in peptide profiles were apparent (Wilks’ Lambda, *F*_66,57.6_ = 1.625, *P* = 0.031; Figure 5b).

## Discussion

We found that both ranaviral disease and chytridiomycosis can contribute to mortality of water frogs in Switzerland. Field surveys have shown that at many sites, *Bd* infection prevalence is high ( [8]; L. R. Davis and U. Tobler unpublished data). However, most frogs that had naturally acquired infections in the field did not show subsequent signs of disease development in the lab. In addition, experimental exposure of frogs to *Bd* zoospores under laboratory conditions ideal for *Bd* growth in culture, did not lead to mortality. Most frogs resisted infection, or tolerated low level infections without developing clinical signs of disease. Thus, we conclude that *P. esculentus* and *P. lessonae* are largely disease resistant, and infection tolerant, as adults, given the strains of *Bd* occurring in Switzerland. These frogs may experience disease-related mortality at more susceptible life-history stages, but if so, such die-offs have not substantially affected population sizes [11]. In addition, 0 of 528 *Pelophylax spp*. tadpoles had detectable *Bd* infections when sampled from 16 ponds in Switzerland including ponds with infected adults [26]. Factors leading to the stable co-existence of host populations and the chytrid fungal pathogen are only beginning to be understood [27]. Both the adaptive and innate branches of immunity are involved [12].

By experimentally reducing two components of innate immunity, antimicrobial skin peptides and skin microbiota, we aimed to determine the relative importance of each defense. However, even when both defenses were experimentally reduced, and the frogs were exposed to repeated infectious doses of *Bd*, we did not detect increased mortality or even an increase in infection intensity. Thus, the effects of reduced immunity and *Bd* were subclinical.

Subclinical effects of *Bd* have been determined in other systems including *Bufo bufo* larvae [28], *Pseudacris regilla* larvae [5], *Plethodon cinereus* adults [16], and *Hyla chrysoscelis* and *Bufo fowleri* metamorphs [29,30]. Here, we found that *Bd* infection and a reduction of both peptide and microbiota immune defenses interacted to produce a growth reduction effect. Our data suggest a trade-off between energy expenditure on growth and immune defense. Given that antimicrobial peptides may be costly to produce, it is not surprising that *P. lessonae* appeared to increase peptide production upon exposure to *Bd* only when microbiota was reduced. Microbiota may extend the host innate immunity against *Bd*[31], thus reducing the need for costly peptide production.

The significant reduction of growth in naturally infected compared to uninfected frogs may indicate cause or consequence of *Bd* infection. When uninfected frogs were experimentally exposed to *Bd* and when they became infected, growth reduction was not simply a result of *Bd* exposure, but an interaction of *Bd* exposure and immune reduction. Thus, factors in addition to natural *Bd* exposure probably contributed to reduced growth in the 14 naturally infected frogs, and these co-factors may have contributed to the susceptibility of these frogs in nature.

There are at least three explanations for why water frogs continued to resist chytridiomycosis upon experimental innate-immune reduction: (1) Host frogs increased synthesis or expression of antimicrobial skin peptide defenses upon exposure to *Bd*. Skin peptides were initially reduced, but when collected again after 64 d, peptide levels had recovered to previous quantities. (2) Some beneficial bacteria may have persisted on skin despite treatments with broad spectrum antibiotics. Ongoing studies will determine the resistance or resilience of amphibian skin microbiota to antibiotic treatments. (3) Water frogs in this study were collected as adults from a population coexisting with ranavirus and *Bd*. Thus, frogs were immunologically primed prior to experimental exposure and may have activated adaptive immune responses. Similarly, wild-caught *Hypsiboas crepitans* resisted infection in one study and may have been immunologically primed [32]. Amphibians in several other studies were able to clear infections at various temperatures including those conducive to fungal growth [rev. in [33]. Although we did not determine the primary source of disease resistance in this study, it is clear that both *Bd* and the maintenance of innate immunity have subclinical costs to water frogs. Colonization by microbiota may help reduce these costs.

We found that the method of infection diagnosis is important for classifying the effects of *Bd* exposure. Although numerous studies have shown that hundreds of amphibian species were positive for *Bd* when sampled ( http://www.Bd-maps.net/), diagnostic PCR to detect *Bd* DNA does not always detect low-level infections, and thus may underestimate infection prevalence. We found that low level infections can be missed by swabbing at a single time point. We also found differences in detectability depending on body location of swabbing, however this is confounded by differences in swabbing date. Other studies have demonstrated higher infection loads on ventral as opposed to dorsal skin surfaces [34]. In addition, DNA of dead fungal cells can be detected, as well as DNA from zoospores in water or that happen to be on the amphibian skin, but not infecting it [35]. This becomes very important for interpreting the effects of experimental exposure to *Bd* when large doses of zoospores are applied. Researchers use several techniques to discriminate between skin infection and transient pathogen presence: repeating diagnostic qPCR from skin swab samples taken over several days with rinses or water changes between sampling periods ( [32,35]; this study), microscopy of skin samples or shed skin [36,37], or histology of skin tissue samples [38]. Detecting the effects of the disease chytridiomycosis is usually determined by clinical signs and histological descriptions of disease state [39]. Diseased frogs exhibit increased skin sloughing, inappetance, lethargy, and loss of righting-reflex [39,40]. These signs usually occur within hours to days of death [41] and indicate a standard endpoint for euthanasia in experimental trials to reduce animal suffering. Only one frog in our study showed clinical signs of chytridiomycosis; unusual skin sloughing was observed for over 2 months before presentation of lethal disease.

## Conclusion

Amphibian species differ in their tolerance of pathogens such as *Bd* or ranavirus [4,10]. Changes in ecological conditions including the microbial communities present in the host environment [42] may influence the immune competence of host amphibians. Intact skin microbiota reduced immune activation and may minimize subclinical costs of infection.

## Methods

### Animal collection and husbandry

We collected seventy water frogs, *Pelophylax lessonae* (= *Rana lessonae*) and *P. esculentus* (= *Rana esculenta*), from a wetland near the Community of Hinwil, Canton of Zürich, Switzerland (47°18’N/8°49’E; 1 km WSW Hinwil, 22 km ESE Zürich) in August 2008. The genetics of this population were previously studied [43,44]. We captured amphibians by hand and immediately placed them into individual sterile plastic boxes. After swabbing and toe-clipping frogs the next day (see below), we placed frogs into new plastic enclosures containing a clay saucer and filter-sterilized tap water. Frogs were fed crickets two to three times weekly after water changes. After acclimation for two weeks, frogs were allocated into one of five treatment regimes and enclosures placed in a randomized block design on shelving in a controlled environment room kept at approximately constant 18°C and 14:10 light:dark schedule with full spectrum lighting. Collecting permits were provided by the Canton Zürich Office of Landscape and Nature conservation directorate, and all animal procedures were approved by the Veterinary Authority for the canton of Zürich (227/2007) and the Federal Office for the Environment.

### Experimental design

We randomly allocated 67 frogs among five treatment regimes without reference to infection status or species (Table 1). To reduce symbiotic microbiota we added antibiotics (10 units*ml^-1^ penicillin, 10 ug*ml^-1^ streptomycin, and 10 ug*ml^-1^ tetracycline; Sigma, St. Louis, Missouri) to the water and filter sterilized before every water change. These doses were deemed safe and effective in other studies ( [45]; L.A. Rollins-Smith pers. comm.). We used filter-sterilized tap water without antibiotics in all other treatments. To reduce antimicrobial skin peptides we administered norepinephrine at a dose that strongly reduces skin peptides in other species [46,47]. All other frogs were given amphibian phosphate-buffered saline as a control for injection. After 64 d, peptides were induced from frogs in all treatments. These final peptide samples were analyzed by mass spectrometry (see below). Exposure to *Bd* zoospores occurred on two successive days beginning the day following peptide reduction. We collected zoospores from a Swiss *Bd* isolate (TG 739) by washing 5 day old 1% tryptone agar plates (grown at 18°C) for 20 min with sterile frog water. We added approximately 1.5 × 10^5^ - 2 × 10^5^ zoospores, or sterile wash for controls, directly onto each frog sitting in 150 ml water. We determined the viability of zoospores by microscopic examination and counted zoospores stained 1:10 in lugol solution (Sigma) on a heamocytometer. Water was changed one day after the final exposure. Frogs were monitored daily during the 64 d experiment.

**Table 1:** **Experimental design including sample size for each water frog species, the type of immune reduction, and exposure to *B. dendrobatidis *zoospores**

**Treatment regime**	**N**	**N**	**Antimicrobial peptide reduced**	**Microbe reduced**	***Bd *exposed**
	***P. lessonae***	***P. esculentus***			
1	5	3	*	*	-
2	10	5			+
3	12	2		*	+
4	8	7	*		+
5	14	1	*	*	+

Treatments began before species determination, therefore, sample sizes vary. * indicates immune reduction treatment.

### Ranavirus analysis

Toe clips from 70 frogs were collected in ethanol and tested for the presence of ranaviral DNA [48]. Two frogs died before the beginning of the experiments and were frozen for later virological testing. Samples from liver, kidney, intestine, lung, and heart were collected in 3 ml Dulbecco’s modified Eagle’s medium (DMEM) (Biochrom AG, Berlin, Germany) supplemented with antibiotics. Cells were disrupted using a Branson-250 Sonifier at an output level of 30 for 3 impulses and cell debris was pelleted by low speed centrifugation (1500×g, 10 minutes at 4°C). The supernatant was used for DNA preparation and virus isolation. For virus isolation, 200 μl of supernatant were inoculated onto iguana heart cells (IgH2, ATCC, CCL-108) following the protocol described elsewhere [49]. Samples showing cytopathic effects (CPE) were passaged onto new IgH2. Virus isolates were characterized by type of CPE and sensitivity to chloroform and detection of ranavirus DNA. Toe clips were transferred into DMEM and treated as described above. DNA was prepared from sample homogenates or cell culture supernatant using the DNAeasy® kit (Qiagen GmbH, Hilden, Germany). A PCR targeting a conserved portion of the major capsid protein (MCP) gene of ranaviruses was carried out as described previously [50]. PCR products were sequenced directly. The sequences were compared to the data in GenBank (National Center for Biotechnology Information, Bethesda, USA) online ( http://www.ncbi.nih.gov) using BLASTN and BLASTX options.

### Bd analysis

We swabbed all frogs at four time points with sterile rayon swabs (Milian). Feet only were swabbed 6 d before the first exposure and at 40 d post exposure. After rinsing with sterile water, we swabbed the body 11 d pre-exposure and 64 d post-exposure. We compared results from both types of swabs. A *Bd* positive result at either pre-exposure time-point was considered infected before the experiment. If a frog had a *Bd* positive result at either post-exposure time-point we considered it infected at the end of the experiment. We extracted DNA from swabs using PrepMan Ultra (Applied Biosystems) and detected *Bd* genomic equivalents with quantitative real-time PCR according to Boyle et al. [51]. Standards of 0.1, 1, 10, and 100 zoospore equivalents were obtained from ecogenics GmbH (Zürich-Schlieren, Switzerland) and run in duplicate. All samples were diluted 1:10 to prevent PrepMan inhibition of the PCR and run in duplicate. When the result was ambivalent, the analysis was repeated. A result below the lowest standard (0.1 zoospore equivalents) was considered negative.

### Species determination

Water frogs form a hybridogenic species complex including the parent species *Pelophylax ridibundus* (R) and *P. lessonae* (L). Hybrid *P. esculentus* (E) can occur with parent species or in all hybrid populations [52]. Since sexual hybrid populations can potentially exist independently of parent species, we here refer to hybrid *P. esculentus* as a “species”. In Switzerland, most water frog populations consist of only *P. lessonae* and *P. esculentus* (LE populations; [44]). These two species can be difficult to distinguish morphologically and microsatellite markers have been identified to distinguish them in northern Europe. We used 16 primer pairs to amplify loci in either the L genome, the R genome, or both, in a multiplex PCR according to Christiansen and Reyer [52] from toe-tip extracted DNA.

### Skin peptide collection and analysis by MALDI-MS

We induced granular gland secretions by administration of norepinephrine (40 nmol/g body mass bitartrate salt, Sigma) by subcutaneous injection [46,47]. After administration of norepinephrine, skin secretions were collected for 15 min in water and acidified with 1% hydrochloric acid to pH < 4.0 to help prevent proteolytic degradation of the samples. Skin secretions were then partially purified by passing over C-18 Sep-Pak cartridges (Waters Corp., Milford, Massachusetts) activated with acetonitrile, eluted in buffer containing 70% acetonitrile and 0.1% trifluoroacetic acid (TFA), spun dry at 60°C, weighed, and stored at −20°C. The total dry weight quantity of partially-purified skin secretions containing peptides recovered per gram body mass (μg gbm^-1^) was determined for each sample.

We analyzed peptide samples collected at day 64 from all frogs by matrix-assisted laser desorption/ionization (MALDI) mass spectrometry (MS) using an Autoflex I time-of-flight mass spectrometer (Bruker Daltonics GmbH, Bremen, Germany) equipped with a 337 nm nitrogen laser. Samples were reconstituted in water and standardized at a concentration of 1 mg ml^-1^. A 5 μl sample solution was diluted with a solution of 10 μl water + 0.1% trifluoroacetic acid containing 10 μg ml^-1^ alytesin (amino acid sequence: pE-GRLGTQWAVGHLM-NH_2_) internal peptide standard (GeneScript). We spotted 1 μl on a “Prespotted AnchorChip” target prepared with α-cyano-4-hydroxycinnamic acid as matrix (HCCA, Bruker), waited 1 min, and rinsed with 7 μl 10 mM aqueous ammonium dihydrogen phosphate buffer containing 0.1% TFA. Instrument calibration was obtained using signals from the HCCA matrix at m/z 379.09 and a mixture of standard peptides composed of Bradykinin 1–7 (m/z 757.40), angiotensin II (m/z 1046.54), angiotensin I (m/z 1296.69), renin substrate (m/z 1758.93), ACTH clip 18–39 (m/z 2465.20) and Somatostatin 28 (m/z 3147.47) all obtained from the peptide calibration standard II mix (Bruker).

For each mass spectrum we calculated the intensity of each peptide peak in proportion to the alytesin external standard. Since peptides may vary in ionization and detectability by MALDI-TOF MS, intensities do not indicate absolute quantities of each peptide. We rank transformed peptide intensity data to satisfy Box’s test for homogeneity of covariance matrices. The mean ranked peptide intensity was calculated for each treatment. We independently explored the effects of various factors on peptide profiles with multivariate analyses of variance including antibiotic treatment, norepinephrine treatment, exposure to *Bd*, and infection with *Bd*. Effects of significant factors were then visualized with a canonical variates analysis (PAST v.2.10 http://folk.uio.no/ohammer/past).

### Statistical analyses

All statistics were carried out using SPSS Statistics 17.0 (SPSS Inc., Chicago, IL, USA). We used standard parametric analyses when the data met the assumptions of normal distribution of data and homogeneity of variances (Levene’s statistic). Otherwise, we used homologous non-parametric tests as indicated.

## Competing interests

The authors declare that they have no competing interests.

## Authors’ contributions

DCW designed and carried out the experiment. LB analyzed skin peptide samples with MALDI mass spectrometry. RM analyzed samples for ranavirus. All authors participated in drafting the manuscript and have read and approved the final manuscript.
